# Myokines as Support Mechanisms for Lung Maintenance and Repair: Implications for Chronic Obstructive Pulmonary Disease

**DOI:** 10.1096/fj.202601931RRRR

**Published:** 2026-11-14

**Authors:** Sven Manse, Ramon C. J. Langen, Irene H. Heijink, Harry R. Gosker

**Affiliations:** ^1^ Department of Respiratory Medicine, NUTRIM Institute for Nutrition and Translational Research in Metabolism Maastricht University Medical Centre+ Maastricht the Netherlands; ^2^ Department of Pathology University Medical Center Groningen, University of Groningen Groningen the Netherlands; ^3^ Department of Medical Biology Pulmonary Diseases University Medical Center Groningen, University of Groningen Groningen the Netherlands; ^4^ Groningen Research Institute for Asthma and COPD (GRIAC) University Medical Center Groningen, University of Groningen Groningen the Netherlands

**Keywords:** lung, lung diseases, myokines, regeneration, secretome, skeletal muscle

## Abstract

Skeletal muscle is increasingly recognized as an active endocrine organ capable of exerting protective effects on distal tissue homeostasis through the secretion of bioactive factors, including myokines. While inter‐tissue communication of skeletal muscle has been described for various tissues, its role in lung biology has remained largely unexplored, though evidence for this interaction is now emerging. Chronic obstructive pulmonary disease, a chronic condition characterized by both pulmonary dysfunction and systemic manifestations such as skeletal muscle wasting, provides a strong rationale to explore the role of the muscle secretome in lung health and pathology. In this review, we explore the concept of skeletal muscle–lung crosstalk with a focus on myokines as key mediators. Through literature search, we selected a subset of myokines for which published studies have specifically investigated their effects on lung disease. We highlight four myokines—irisin, apelin, MG53, and Metrnl—that have demonstrated protective effects on lung tissue across a range of experimental models. These factors exert anti‐inflammatory, antioxidative, antifibrotic, and cytoprotective effects by modulating key signaling pathways. Through these mechanisms, myokines contribute to the preservation of lung tissue integrity, regulation of inflammation in the lungs, maintenance of lung mitochondrial function, and support of lung tissue repair processes. Collectively, these findings support a paradigm in which skeletal muscle actively contributes to lung health. Elucidating this crosstalk may reveal novel strategies to enhance pulmonary resilience and repair, particularly through interventions that deploy the muscle secretome.

## Graphical abstract

Release of myokines, including irisin, apelin, MG53, and Metrnl, can be stimulated by exercise interventions. The release of these myokines has a beneficial impact on lung health by inhibiting various pathophysiological pathways implicated in lung disease, such as inflammation, oxidative stress, mitochondrial stress, and excessive cell death. At the same time, these myokines stimulate cell repair mechanisms. Collectively, this suggests that skeletal muscle can contribute to lung health via the release of myokines.

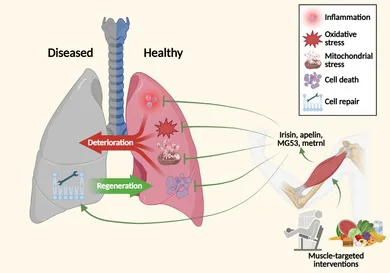

## 1 Introduction

Chronic obstructive pulmonary disease (COPD) is a progressive lung disease characterized by mucus hypersecretion (chronic bronchitis), (small) airway remodeling and alveolar destruction (emphysema), resulting in airflow limitation and accelerated pulmonary decline [1]. COPD is the third leading cause of death worldwide and affects over 390 million people globally, a number which is expected to rise with the aging of our population [2].

The symptoms and pathophysiology of COPD are not limited to the lungs and involve other tissues as well, including skeletal muscle, evidenced by muscle wasting. Muscle wasting has been proposed to be a passive, systemic manifestation of the pulmonary disease process in response to an overspill of inflammatory mediators. On the other hand, it could reflect an adaptive mechanism to support lung tissue health or functional recovery, resulting in muscle tissue depletion as an evolutionary preserved process to redirect organismal amino acid pools. Furthermore, loss of muscle function may contribute to the pathological process in the lungs, while exercise and/or a healthy condition of the muscles may even be required to support lung health. Thus, muscle‐targeted therapies may have beneficial effects on lung health. We will discuss these concepts in this review.

A list of candidate myokines was first identified from the literature and subsequently evaluated based on the relevance of skeletal muscle as a predominant source. Candidate myokines were then screened for experimental evidence of effects on lung tissue. Studies focusing primarily on lung cancer were excluded, as malignant disease involves distinct pathophysiological mechanisms and falls outside the scope of this review. This process resulted in the selection of irisin, apelin, MG53 and Metrnl for detailed discussion. For each of these myokines, a targeted PubMed search was performed using myokine‐specific and pulmonary search terms and a manual screening was performed. The exact search strings are provided in Supplementary Table S1.

## 2 COPD Pathophysiology

The main causes of lung pathology are exposure to cigarette smoke or inhalation of other noxious particles. These inflict oxidative damage to airway epithelia, resulting in cellular stress and mitochondrial dysfunction. Not all smokers develop COPD, and especially when activation of antioxidant defenses like the Nrf2‐HO‐1 axis is insufficient, this limits the capacity to neutralize reactive oxygen species (ROS) [3, 4]. This leads to the activation of inflammatory cascades [5], mediated through activation of pathways such as NF‐κB [6] and the NLRP3 inflammasome [7]. Consequently, various immune cell types are recruited, such as elastase‐releasing neutrophils, further contributing to the destruction of alveolar walls, progressive airway narrowing, and excessive mucus production [8, 9, 10]. In COPD lungs, this simultaneously disrupts mechanisms required for tissue recovery. Abnormal lung epithelial repair mechanisms have been observed in COPD, which in the small airways may be driven by increased TGF‐β signaling, leading to airway wall thickening [11]. The abnormal inflammatory and repair processes may result in an increased burden of oxidative stress and mitochondrial dysfunction [12]. Mitochondrial abnormalities, including reduced mitochondrial respiration [13, 14], increased ROS production, and dysregulated mitophagy have been observed in structural lung cells from COPD patients. Mitochondrial dysfunction has been shown to be involved in processes related to not only pro‐inflammatory responses but also apoptosis, cellular senescence, exhaustion of stem cells and impaired lung epithelial repair, and may thus further contribute to disease progression [14, 15, 16, 17]. The persistent interplay of oxidative stress and inflammation in COPD further contributes to dysfunction of repair mechanisms [18, 19] and contributes to stem cell exhaustion, characterized by the accumulation of senescent tissue‐resident stem cells, such as epithelial stem cells [20], thereby impairing tissue regeneration and promoting disease progression. In addition to inflammation and structural remodeling, COPD is increasingly associated with metabolic alterations within lung‐resident cells, including mitochondrial dysfunction, altered redox balance and changes in glycolytic activity [16, 21, 22]. These metabolic changes may contribute to persistent inflammatory activation and impaired epithelial repair.

Besides the pulmonary manifestations, patients with COPD often experience skeletal muscle wasting, characterized by a reduction in skeletal muscle mass and strength, as well as loss of oxidative metabolic and endurance capacity [23, 24, 25]. Low skeletal muscle mass has been correlated with disease progression, exacerbation frequency, and reduced quality of life [26, 27, 28]. Skeletal muscle wasting in COPD may be caused by excess pulmonary inflammation spilling over into the circulatory system, ultimately affecting skeletal muscle health, a theory also known as the “spill‐over theory”. Skeletal muscle functions as the body's largest protein reservoir and plays a key role in systemic amino acid homeostasis. During catabolic stress in disease, skeletal muscle releases amino acids such as alanine and glutamine to support systemic demands [29, 30, 31]. Accordingly, muscle wasting may not just be a consequence of disease, but rather an adaptive response in which skeletal muscle‐derived amino acids are mobilized to sustain whole‐body homeostasis. The notion that alterations in skeletal muscle have a systemic impact also extends to training‐induced changes in muscle metabolism in health and disease [32, 33], including lung disease. Hence, therapies targeted to improve muscle health may prove beneficial in improving lung health.

Interestingly, a meta‐analysis of randomized controlled trials showed that whole‐body exercise training consistently improved pulmonary function in COPD [34]. A three‐year study found that exercise significantly slowed the rate of forced expiratory volume in 1 s (FEV_1_) decline in patients undergoing exercise training compared to non‐exercised patients [35]. This effect was confirmed in a different study, where FEV_1_ even remained stable in patients undergoing exercise intervention over the course of three years compared to the control group [36]. Exercise training furthermore seems to reduce exacerbation frequency [37]. Moreover, aerobic exercise stimulated lung regeneration by promoting bronchioloalveolar stem cell expansion in a mouse model [38]. These observations suggest that communication between the lungs and skeletal muscle in pulmonary disease may extend beyond the mono‐directional “spill‐over” theory resulting in muscle dysfunction, and implicate an additional, reverse directionality in which skeletal muscle may contribute to lung health.

Evidence shows that, besides amino acids, other skeletal muscle‐derived biomolecules can contribute to homeostasis of distant organs. Factors actively released from contracting muscle (i.e., after exercise) can modulate systemic metabolism, influence immune cell function and alter signaling pathways in distant tissues. For example, in mice, muscle‐derived mediators have been shown to regulate hepatic glucose production, promote lipid oxidation and improve insulin sensitivity in metabolic tissues, such as the liver [39, 40] and attenuate renal fibrosis [41]. Communication between muscle and the cardiovascular system contributes to vascular and cardiac integrity, while muscle‐driven signals are also positively implicated in neuroplasticity and cognitive function [42, 43, 44, 45]. Interestingly though, direct evidence for the impact of skeletal muscle‐derived factors on pulmonary health is lacking. In this review, we explore the possible mediators via which skeletal muscle can benefit lung homeostasis, specifically focusing on secreted ligands acting on mechanisms relevant in the context of chronic respiratory diseases like COPD.

## 3 Effects of Myokines on Lung Health

Skeletal muscle is increasingly gaining attention as an active secretory organ. It can release a wide range of cytokines, peptides and proteins, also known as myokines. These myokines can be secreted by muscle fibers in response to contraction following exercise and are therefore often called exerkines [40, 46]. Myokines have autocrine, paracrine and endocrine functions and can play critical roles in regulating metabolic processes and inflammation in distant tissues such as the liver, adipose tissue and the brain [40, 44, 47]. Notably, various myokines, including irisin, apelin, MG53 and Metrnl have been shown to affect health of lung cells both in vitro and in vivo, suggesting a myokine‐based skeletal muscle‐lung crosstalk that may influence pulmonary stress and injury responses.

### 3.1 Irisin/FNDC5

Irisin, first identified by Boström et al. in 2012, is a cleaved fragment of its membrane‐bound precursor fibronectin type III domain‐containing 5 (FNDC5) and is secreted as a circulating myokine following muscle contraction [48]. Irisin binds to αV‐containing integrins and activates various pathways, such as the MAPK, AMPK, PGC‐1α and PI3K‐AKT axes [49]. Initially described as a mediator of white‐to‐brown adipose tissue conversion through induction of mitochondrial uncoupling protein 1 (UCP‐1), irisin promotes thermogenesis and energy expenditure in adipose tissue. Subsequent studies revealed that irisin also acts on distant organs, including the lungs, where it exerts protective effects against inflammation and oxidative stress [48].

Evidence for the beneficial role of irisin in lung pathology has been provided in both clinical and experimental settings. In a mouse model of cigarette smoke‐induced COPD, concurrent exercise training elevated irisin levels, and attenuated alveolar destruction and neutrophil infiltration in bronchoalveolar lavage fluid [50]. Lung‐protective effects of irisin have further been demonstrated in models of acute lung injury (ALI). In sepsis‐induced ALI, irisin administration reduced immune cell infiltration in lung tissue, alveolar and interstitial edema, and lung epithelial apoptosis, while restoring barrier integrity through upregulation of tight junction proteins [51, 52]. Irisin‐induced activation of the Nrf2–HO‐1 axis has also been reported in hyperoxia‐induced bronchopulmonary dysplasia, further supporting its antioxidative effects in the lung [53]. In addition, irisin pretreatment reduced systemic inflammation and lung injury induced by fine particles in mice [54]. Finally, irisin has been shown to protect from ischemia/reperfusion lung damage in a mouse model via UCP2‐mediated preservation of mitochondrial function [55].

The molecular mechanisms reported to be involved in these lung‐protective effects include multiple intracellular signaling pathways. In cigarette smoke‐induced emphysema and hyperoxic lung injury, irisin increased Nrf2 and HO‐1 expression [50, 53]. In LPS‐stimulated A549 cells, irisin inhibited NF‐κB and caspase‐3 protein expression via activation of the AMPK/SIRT1 pathway, and inhibition of AMPK reversed these effects, supporting a role for AMPK in irisin‐mediated protection [51]. In fine particle‐induced ALI mice, irisin restored autophagic flux in alveolar macrophages through activation of the AMPK/mTOR pathway and increased expression of autophagy‐related proteins [54]. In addition, irisin inhibited NLRP3 inflammasome activation and reduced secretion of IL‐1β, IL‐18, and TNF‐α in alveolar macrophages by blocking the Nod2/NF‐κB pathway [56], and promoted macrophage polarization toward an anti‐inflammatory phenotype via activation of JAK2–STAT6 signaling and subsequent induction of PPARγ and Nrf2 [57].

### 3.2 Apelin

Apelin is an endogenous peptide synthesized as a 77‐amino acid precursor that undergoes post‐translational processing to generate shorter, active derivatives that are named after the number of amino acids they contain. Of these derivatives, the 13 amino acid long apelin‐13 is most studied due to its strong biological activity and stability. Apelin peptides act as ligands for the G protein‐coupled receptor APJ, which is involved in cellular metabolism and is widely expressed across tissues, including the lungs, though a direct relation between APJ activation and cellular repair mechanisms is underexplored. Although not restricted to skeletal muscle, apelin has been proposed as a myokine following the observation that circulating apelin levels increase after exercise training in a cohort of patients with obesity and that cultured myotubes release apelin in response to exercise‐mimicking stimuli in vitro [58].

A growing body of evidence supports a beneficial role for apelin in lung recovery and structural preservation. Apelin has consistently been found to attenuate lung fibrosis in both in vitro and in vivo models of LPS‐induced and silicosis‐associated pulmonary fibrosis. In these models, apelin‐13 administration reduced collagen deposition and alveolar septal thickening and was associated with improved lung function parameters [59, 60, 61]. Apelin‐13 was effective both prior to and after onset of lung fibrosis, indicating its potential not only to prevent but also to reverse ongoing fibrotic remodeling [61]. In line with these findings, serum apelin levels were associated with improved forced vital capacity (FVC) percentage and FEV_1_ percentage in patients with silicosis [61]. Beyond fibrotic disease, apelin also improved lung outcomes in models of ALI and ventilation‐induced lung injury (VILI); in LPS‐induced ALI, apelin‐13 improved endothelial and epithelial barrier integrity [62, 63], while apelin‐36 reduced lung edema and epithelial cell apoptosis in rat models [64]. In VILI, apelin‐13 mitigated lung edema and tissue damage and reduced oxidative stress markers [65].

These lung‐protective effects are mediated through several intracellular signaling pathways. In pulmonary fibrosis models, apelin‐13 suppressed expression of markers of endothelial‐to‐mesenchymal transition via enhanced angiotensin‐converting enzyme 2 (ACE2) expression and inhibition of the TGF‐β1/SMAD signaling pathway [59, 60, 61]. In lung lysates LPS‐induced ALI mouse models, apelin promoted mitochondrial biogenesis, decreased mitochondrial degradation, and restored autophagic flux through stimulation of AMPK signaling, thereby preserving barrier integrity [62, 63]. Apelin also prevented DNA damage in epithelial cells, preserving epithelial integrity [63]. In parallel, apelin‐36 inhibited epithelial apoptosis through activation of MAPK signaling [64]. In VILI, apelin shifted signaling toward anti‐apoptotic pathways by increasing Bcl‐2 and reducing Bax expression and decreased oxidative stress by lowering myeloperoxidase and malondialdehyde levels while increasing catalase activity [65]. Inflammatory modulation further contributed to these effects, as apelin‐13 attenuated LPS‐induced pro‐inflammatory cytokine secretion in macrophages and reduced TNF‐α, IL‐1β, and IL‐6 levels in BALF in models of ALI and VILI [64, 65, 66].

### 3.3 MG53/TRIM72

Mitsugumin 53 (MG53), also known as TRIM72, is a member of the multifunctional tripartite motif family and is mainly expressed in striated muscle, including skeletal muscle. MG53 interacts with phosphatidylserine in damaged membranes to facilitate intracellular vesicle trafficking to the damaged site in skeletal muscle [67], though the molecular interactions in lung tissue remain unclear. Elevated MG53 levels have been observed in murine models after treadmill exercise, and glucose and insulin induce the release of MG53 from isolated skeletal muscle, confirming its role as a myokine [68, 69].

In rat models of ischemia–reperfusion injury, elastase‐induced emphysema, and LPS‐induced acute lung injury, administration of MG53 via intravenous or intratracheal routes reduced lung cell death, inflammation, pulmonary edema, and alveolar destruction while improving blood oxygenation [70]. MG53 also enhanced resistance of alveolar epithelial cells to mechanical injury and promoted repair after damage, reducing LDH release and cell death in ventilation‐injured lungs both in vitro and in vivo [71, 72]. Beyond acute injury, MG53 exerted protective effects in models of chronic pulmonary stress: In asthma, MG53 suppressed airway inflammation, immune cell infiltration, mucus production, and type II cytokine expression [73]. MG53 further prevented fibrotic remodeling by maintaining alveolar epithelial integrity and inhibiting apoptosis in chronic stress conditions [74]. MG53 deficiency aggravated ischemia‐induced endothelial injury and graft damage, whereas MG53 overexpression preserved lung structure and reduced apoptosis in the context of lung transplantation [75]. Mechanistically, MG53 supports lung resilience primarily through preservation of membrane integrity. MG53 modulates caveolar endocytosis, a crucial step in cell membrane repair. MG53 knockout reduced membrane localization of caveolin‐1 and impaired endocytosis, resulting in exacerbated lung damage after mechanical stress, whereas MG53 overexpression restored these processes [76]. In addition, MG53 suppressed NF‐κB signaling in models of airway inflammation [73] and inhibited apoptosis partly through modulation of p53 activation [74]. MG53 also preserved endothelial integrity during ischemic stress in transplanted lungs [75]. MG53 has been implicated in muscle–lung crosstalk in chronic disease. In COPD, plasma MG53 levels in sarcopenic patients were reduced compared to non‐sarcopenic patients, and MG53 deficiency aggravated cigarette smoke‐induced muscle wasting and disrupted mitochondrial fission through loss of interaction with cardiolipin [77].

### 3.4 Meteorin‐Like/Metrnl

Meteorin‐like, also known as meteorin‐β, Metrnl, cometin, or subfatin, is a secreted protein induced by exercise and has emerged as a potential myokine. Metrnl binds to the receptor tyrosine kinase (KIT) and is known to activate pathways such as AMPK, PPARδ and PGC‐1α [78, 79]. It was initially identified as a circulating factor induced by PGC‐1α in muscle and cold exposure in fat, and subsequent studies confirmed that skeletal muscle secretes Metrnl in response to contraction [80]. Metrnl is known to modulate anti‐inflammatory immune responses and tissue remodeling, suggesting a potential role in lung repair and homeostasis [81].

Evidence supporting a protective role for Metrnl in lung pathology has been obtained across multiple disease models. In a mouse model of bleomycin‐induced idiopathic pulmonary fibrosis, Metrnl expression was reduced in lung tissue, while its negative regulator miR‐410‐3p was upregulated [82]. Restoration of Metrnl signaling alleviated TGF‐β1‐induced oxidative stress, inflammation, and fibrosis, as reflected by reduced ROS levels, decreased pro‐inflammatory TNF‐α, IL‐1β, and IL‐6 expression, and downregulation of fibrotic markers α‐SMA and collagen‐I and‐III [82]. In models of acute lung injury, Metrnl overexpression reduced LPS‐induced lung inflammation and ferroptosis, whereas knockdown exacerbated injury [83]. Metrnl administration decreased inflammatory cell infiltration and lung edema and suppressed NF‐κB activation in mice, while protecting bronchial epithelial cells from ROS accumulation and cytokine secretion in vitro [84]. Metrnl also exerted anti‐inflammatory effects in allergic airway disease. In house dust mite‐induced asthma, Metrnl levels were elevated in serum, BALF, and lung tissue, and its administration reduced immune cell infiltration, Th2‐type inflammation, and airway hyperresponsiveness [85]. Similar protective effects were observed in ovalbumin/LPS‐induced allergic airway inflammation, where Metrnl reduced leukocyte and neutrophil infiltration, oxidative stress, and pro‐inflammatory cytokine expression [86].

These effects are mediated through coordinated regulation of metabolic and inflammatory signaling pathways. In pulmonary fibrosis, Metrnl activated the AMPK/SIRT1/NFκB signaling axis, thereby attenuating oxidative and inflammatory injury [82]. In ALI, Metrnl reduced ferroptosis through the SIRT1–p53–SLC7A11 pathway and suppressed NFκB activation while increasing AMPK phosphorylation and SIRT1 expression [83, 84]. In allergic airway inflammation, Metrnl inhibited the IKK/IκB/NFκB pathway, contributing to reduced inflammatory and profibrotic signaling [86].

### 3.5 Other Myokines That May Modulate Lung Health Related Processes

Other circulating mediators, including adiponectin, leptin and interleukins, have been implicated in the regulation of pulmonary inflammation and health, suggesting that they may impact on to lung homeostasis and repair in COPD and other pulmonary diseases [87, 88, 89, 90, 91]. Adiponectin and leptin are well‐known adipokines being mainly secreted by adipose tissue. The contribution of skeletal muscle to their circulatory levels is therefore relatively small [92, 93]. In COPD, these adipokines have been particularly linked to systemic metabolic alterations, body composition, cachexia and skeletal muscle dysfunction, indicating that their role may be more reflective of adipose–muscle and systemic inflammatory crosstalk than direct skeletal muscle‐to‐lung signaling [94, 95, 96]. Although IL‐6 can promote epithelial regeneration and protect against acute lung injury [89, 90], only a relatively small portion of its circulatory pool is released into the circulation by contracting skeletal muscle [97, 98, 99, 100]. Therefore, while other myokines like adiponectin, leptin and interleukins may contribute to lung‐protective signaling, they are not further discussed in detail in this review.

### 3.6 Mechanistic Overlap and Pathway Convergence

A central recurring feature is the modulation of cytoprotective metabolic and inflammatory signaling. Irisin, apelin and Metrnl have all been linked to AMPK/SIRT1‐related pathways [51, 62, 63, 82, 84], while irisin additionally activates Nrf2–HO‐1 signaling [50, 53] and modulates AMPK/mTOR‐dependent autophagy [54]. Apelin promotes mitochondrial homeostasis and autophagic flux through AMPK signaling [62, 63], whereas Metrnl suppresses inflammatory and oxidative stress responses through AMPK/SIRT1/NF‐κB signaling and regulation of the SIRT1–p53–SLC7A11 axis [82, 83, 84]. MG53 is additionally responsive to metabolic cues such as glucose and insulin [69]. A further area of overlap is immunomodulation and preservation of lung structural integrity. Irisin, apelin and Metrnl suppress pro‐inflammatory signaling, particularly through modulation of NF‐κB [56, 57, 64, 65, 66, 83, 86], while irisin additionally inhibits NLRP3 inflammasome activation and promotes anti‐inflammatory macrophage polarization through JAK2–STAT6, PPARγ and Nrf2 signaling [56, 57]. In parallel, irisin and apelin support epithelial or endothelial barrier integrity [52, 62, 63, 64, 65], whereas MG53 promotes membrane repair through caveolin‐1‐dependent processes and limits cell death [71, 72, 76]. Apelin inhibits TGF‐β/SMAD‐associated remodeling [59, 61], while Metrnl reduces oxidative stress and fibrotic marker expression [82] and MG53 preserves alveolar epithelial integrity under chronic stress [74]. These pathways intersect with core features of COPD pathophysiology, including oxidative stress, mitochondrial dysfunction, impaired cellular quality control, persistent inflammatory activation and longer‐term structural remodeling of the lung.

The apparent convergence on these pathways should be interpreted with caution. AMPK/SIRT1, Nrf2 and NF‐κB are broadly involved in cellular stress responses, and their modulation by different myokines does not necessarily indicate a shared upstream mechanism or functional redundancy. Rather, distinct receptor systems may independently converge on similar downstream responses. Moreover, pathway involvement has generally been demonstrated in differing cell types and injury models, limiting direct comparison between myokines. Whether their effects are additive, redundant or context‐dependent therefore remains unclear.

## 4 Exercise and Nutritional Regulation of Myokine Expression

Evidence from human exercise studies indicates that irisin, apelin, and Metrnl are responsive to contractile activity, although the degree of evidence differs between myokines. Acute exercise transiently increases circulating irisin in healthy individuals [101, 102, 103], and intensive interval training can increase skeletal muscle FNDC5 expression, the precursor of irisin [104]. Aerobic training and acute high‐intensity exercise have similarly been shown to increase circulating apelin in healthy adults [105, 106], and endurance training increased skeletal muscle apelin expression in men with obesity [58]. For Metrnl, acute and repeated high‐intensity exercise increased skeletal muscle METRNL mRNA expression in healthy men [104], while acute downhill running increased circulating Metrnl levels [107]. In contrast, evidence for exercise‐induced MG53 regulation remains limited to inconsistent preclinical findings, often involving muscle damage or disease models [68, 108, 109]. Collectively, these studies support the exercise responsiveness of irisin, apelin, and Metrnl, whereas comparable evidence for MG53 remains insufficient.

Beyond contractile activity, nutritional and metabolic interventions may also modulate the expression or circulating availability of these myokines, although the available evidence is rather limited. Omega‐3 fatty acid and vitamin D supplementation increased circulating irisin in patients with coronary artery disease, Type 2 diabetes, or subclinical hypothyroidism [110, 111, 112, 113], while a very‐low‐calorie ketogenic diet transiently increased circulating irisin in individuals with overweight or obesity [114]. Dietary weight loss transiently increased circulating Metrnl in individuals with severe obesity [115]. Evidence for direct metabolic regulation of MG53 is restricted mainly to acute glucose‐ and insulin‐stimulated release from skeletal muscle [69].

These human observations are further supported by mechanistic preclinical and in vitro studies showing that skeletal muscle responds to exercise‐ and nutrition‐associated signals by increasing myokine expression and secretion. For example, exercise elevates skeletal muscle expression of the irisin precursor FNDC5 together with circulating irisin levels in mice [116], while cultured myotubes release apelin [58] and increase Metrnl secretion following exercise‐mimicking stimuli [117]. The involvement of exercise‐responsive transcriptional pathways is further supported by a study showing that transgenic expression of PGC‐1α4 stimulates Metrnl mRNA expression and protein secretion, and that an 8‐week exercise program increases skeletal muscle PGC‐1α expression together with circulating Metrnl levels in obese mice [81]. Nutritional compound sesamin increased expression of irisin‐precursor FNDC5 and irisin secretion in C2C12 myotubes [118]. Vitamin D3 supplementation enhanced skeletal muscle apelin expression in aged mice and eicosapentaenoic acid increased apelin expression and secretion in both high‐fat diet‐fed mice and C2C12 myotubes [119, 120]. In addition, metabolic signals such as glucose and insulin stimulate MG53 release from skeletal muscle in dystrophic and diabetic mice [68, 69]. Together, these findings indicate that irisin, apelin, MG53, and Metrnl are part of a broader network of lifestyle‐responsive myokines whose systemic availability is shaped by exercise‐induced skeletal muscle activity and further modulated by nutritional and metabolic status.

## 5 Implications for COPD

As indicated above, irisin, apelin, MG53 and Metrnl act via various lung‐protective pathways that are relevant to COPD pathophysiology. Their general characteristics and potential relevance to COPD are compared in Table 1, while representative studies, disease phenotypes, experimental models and pulmonary outcomes are summarized in Table 2. Although these myokines differ in receptor biology and downstream molecular pathways, their reported actions partly overlap at the level of oxidative stress control, suppression of inflammation, mitochondrial and metabolic resilience, epithelial–endothelial barrier preservation, and cell death regulatory pathways, as shown in Figure 1. However, the evidence presented here is derived from a heterogeneous set of studies, including multiple disease phenotypes and experimental models (Table 2), and extrapolation of this evidence to COPD requires further experimental confirmation.

**TABLE 1:** Skeletal muscle‐derived myokines of lung maintenance and repair.

Myokine	Signaling pathways	Effects on lung	Potential COPD impact
Irisin	AMPK/SIRT1, Nrf2‐HO1, AMPK/mTOR, NF‐κB, NRLP3, JAK2‐STAT6, PPARγ	Reduced oxidative stress and inflammation; enhanced barrier integrity and autophagy	Reduced emphysema progression; enhanced stress resilience
Apelin	AMPK, ACE2, TGF‐β/SMAD, MAPK, Bcl‐2/Bax	Enhanced integrity; reduced apoptosis, oxidative stress and fibrosis	Reduced remodeling; improved vascular function
MG53	Caveolin‐1, NF‐κB, p53	Enhanced membrane repair and tissue integrity; reduced cell death and inflammation	Enhanced repair capacity; reduced injury susceptibility
Metrnl	AMPK/SIRT1, NF‐κB, IKK/IκB, PPARδ, PGC‐1α	Reduced inflammation, oxidative stress, ferroptosis and fibrosis	Reduced chronic inflammation and remodeling

*Note:* Overview of the major signaling pathways, lung‐protective effects and potential COPD relevance of the exercise‐responsive myokines irisin, apelin, MG53 and Metrnl. Despite distinct molecular mechanisms, these myokines overlap on downstream cellular processes relevant to COPD, including regulation of inflammation, oxidative stress, epithelial and endothelial integrity, tissue repair and structural remodeling.

Abbreviations: ACE2, angiotensin‐converting enzyme 2; AMPK, AMP‐activated protein kinase; Bax, Bcl‐2‐associated X protein; Bcl‐2, B‐cell lymphoma 2; HO‐1, heme oxygenase 1; IKK, inhibitor of κB kinase; IκB, inhibitor of nuclear factor κB; JAK2, Janus kinase 2; MAPK, mitogen‐activated protein kinase; mTOR, mechanistic target of rapamycin; NF‐κB, nuclear factor kappa B; NLRP3, NOD‐, LRR‐ and pyrin domain‐containing protein 3; Nrf2, nuclear factor erythroid 2‐related factor 2; PGC‐1α, peroxisome proliferator‐activated receptor gamma coactivator 1‐alpha; PPARδ, peroxisome proliferator‐activated receptor delta; PPARγ, peroxisome proliferator‐activated receptor gamma; SIRT1, sirtuin 1; SMAD, small mothers against decapentaplegic; STAT6, signal transducer and activator of transcription 6; TGF‐β, transforming growth factor beta.

**TABLE 2:** Representative studies investigating the pulmonary effects of skeletal muscle‐derived myokines.

Myokine	Highlighted key study	Disease	Model	Main mechanism(s)	Key pulmonary finding
Irisin	Sugiyama et al. (2017) [121]	COPD‐associated emphysema	GOLD II‐III patient cohort; CSE‐exposed A549 cells	Nrf2‐mediated protection from apoptosis	Lower circulating irisin was associated with greater emphysematous destruction; irisin reduced CSE‐induced epithelial apoptosis
	Kubo et al. (2019) [50]	Cigarette smoke‐induced COPD	CS‐exposed, treadmill‐trained mice	Nrf2‐HO‐1 antioxidant signaling	Exercise‐associated irisin induction reduced alveolar destruction and BALF neutrophil count
	Li et al. (2019) [51]	Sepsis‐induced ALI	ALI rat model; LPS‐stimulated A549 cells	AMPK/SIRT1	Irisin reduced inflammation, edema and epithelial apoptosis
Apelin	Shen et al. (2023) [61]	Silicosis‐associated pulmonary fibrosis	Silicosis patient cohort; silica‐instilled mice; THP‐1 macrophage coculture with MRC‐5 fibroblasts	TGF‐β1‐SMAD inhibition	Apelin prevented and partly reversed collagen deposition, fibroblast activation and alveolar structural damage
	Kong et al. (2021) [62]	LPS‐induced ALI	LPS‐exposed mice and HUVECs	AMPK‐mediated mitochondrial biogenesis and autophagy	Apelin preserved the endothelial barrier and improved mitochondrial function
	Lian et al. (2024) [65]	Ventilator‐induced lung injury	High tidal volume‐ventilated rats	APJ‐Akt and Bcl‐2‐Bax signaling	Apelin reduced lung‐tissue damage, edema, inflammation, oxidative stress and apoptosis
MG53	Jia et al. (2014) [70]	ALI, ischemia–reperfusion injury and emphysema	MG53^−/−^ mice, ischemia–reperfusion and elastase‐injured rats; RLE and A549 cells	MG53‐mediated membrane repair	MG53 reduced epithelial injury, inflammation, edema and alveolar destruction while improving oxygenation
	Cong et al. (2020) [74]	Pulmonary fibrosis	Bleomycin‐exposed mice; primary mouse ATII cells and ATII rat cell‐line	Membrane repair and p53 inhibition	MG53 preserved alveolar epithelial integrity and reduced ATII‐cell apoptosis and pulmonary fibrosis
	Tan et al. (2023) [73]	Allergic asthma	Ovalbumin/alum‐sensitized mice	NF‐κB inhibition	MG53 reduced inflammatory cell infiltration, mucus production, Type 2 cytokines and airway remodeling
Metrnl	Gu et al. (2025) [82]	Pulmonary fibrosis	Bleomycin‐exposed mice; TGF‐β1‐stimulated MRC‐5 cells	AMPK/SIRT1/NF‐κB	Metrnl‐signaling reduced oxidative stress, inflammatory cytokines and expression of collagen I/III
	Chen et al. (2023) [83]	LPS‐induced ALI	LPS‐exposed mice with Metrnl‐overexpression or knockdown; MLE‐12 cells	SIRT1‐p53‐SLC7A11‐mediated ferroptosis inhibition	Metrnl reduced lung injury, inflammation and ferroptosis
	Gao et al. (2022) [85]	Allergic asthma	Asthma patient cohort; house dust mite‐exposed mice; bone marrow‐derived dendritic cells and 3D airway cultures	Suppression of dendritic cell‐mediated Th2 responses	Metrnl reduced airway hyperresponsiveness, inflammatory cell infiltration and Type 2 cytokine production

*Note:* Clinical, in vivo and in vitro studies evaluating irisin, apelin, MG53 and Metrnl are summarized according to disease context, experimental model, principal mechanism and key pulmonary finding.

Abbreviations: 3D, three‐dimensional; ALI, acute lung injury; AMPK, AMP‐activated protein kinase; APJ, apelin receptor; ATII, alveolar type II; BALF, bronchoalveolar lavage fluid; Bax, Bcl‐2‐associated X protein; Bcl‐2, B‐cell lymphoma 2; COPD, chronic obstructive pulmonary disease; CS, cigarette smoke; CSE, cigarette smoke extract; GOLD, Global Initiative for Chronic Obstructive Lung Disease; HDM, house dust mite; HO‐1, heme oxygenase 1; HUVEC, human umbilical vein endothelial cell; LPS, lipopolysaccharide; MG53, mitsugumin 53; MLE‐12, mouse lung epithelial‐12; MRC‐5, human fetal lung fibroblast cell line; NF‐κB, nuclear factor kappa B; Nrf2, nuclear factor erythroid 2‐related factor 2; OVA, ovalbumin; RLE, rat lung epithelial; SIRT1, sirtuin 1; SLC7A11, solute carrier family 7 member 11; TGF‐β1, transforming growth factor beta 1; Th2, T helper Type 2; THP‐1, human monocytic leukemia cell line.

**FIGURE 1:**
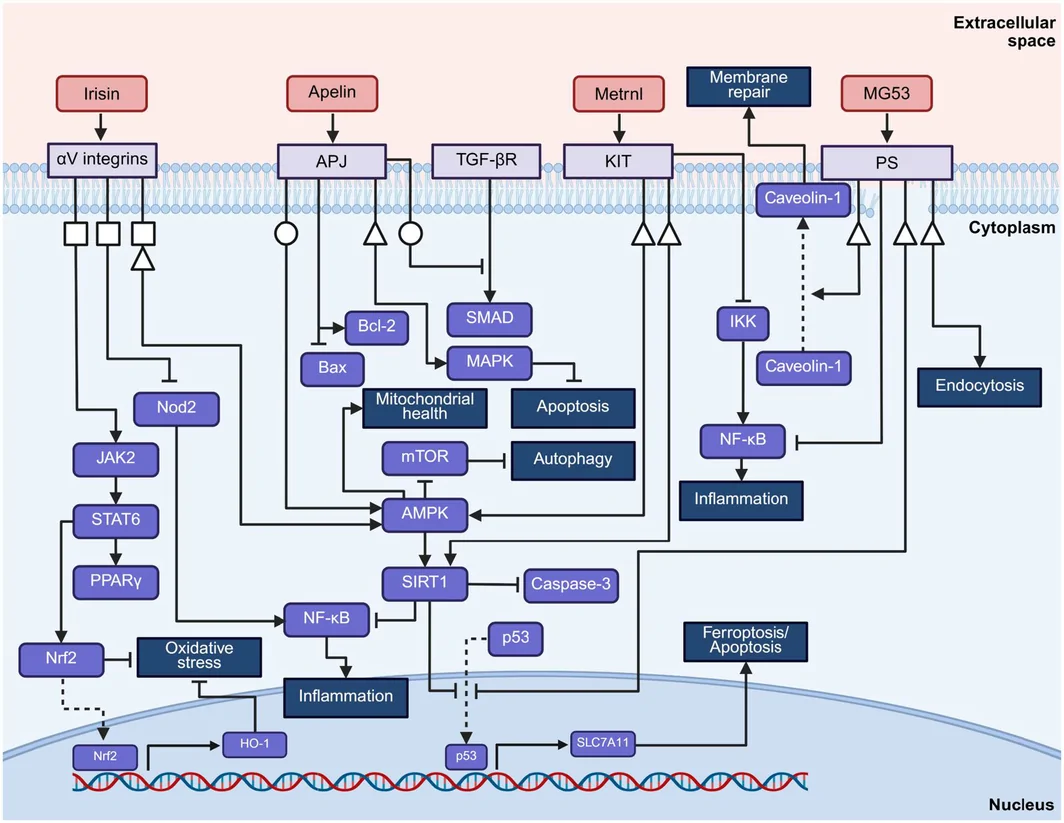
Myokine‐mediated signaling pathways controlling cellular processes involved in lung pathology. Schematic overview of putative lung‐protective signaling pathways modulated by the myokines irisin, apelin, Metrnl and MG53. Myokines are indicated in pink boxes, receptors in gray boxes, intracellular signaling mediators in purple boxes and downstream cellular effects in dark blue boxes. Cell type symbols along signaling branches indicate the cell type in which modulation of the corresponding pathway by the respective myokine has been demonstrated experimentally (Square, macrophage; Circle, endothelial cell; Triangle, epithelial cell). Absence of a symbol indicates that the corresponding evidence was not resolved to one of these specific cell types (e.g., in whole‐lung tissue). Arrows indicate activation or induction, blunt‐ended lines indicate inhibition and dashed arrows indicate translocation events. AMPK, AMP‐activated protein kinase; Bax, Bcl‐2‐associated X protein; Bcl‐2, B‐cell lymphoma 2; HO‐1, heme oxygenase 1; IKK, inhibitor of κB kinase; JAK2, Janus kinase 2; MAPK, mitogen‐activated protein kinase; Metrnl, Meteorin‐like; MG53, mitsugumin 53; mTOR, mechanistic target of rapamycin; NF‐κB, nuclear factor kappa B; NOD2, nucleotide‐binding oligomerization domain‐containing protein 2; Nrf2, nuclear factor erythroid 2‐related factor 2; p53, tumor protein p53; PPAR, peroxisome proliferator‐activated receptor; SIRT1, sirtuin 1; SLC7A11, solute carrier family 7 member 11; SMAD, small mothers against decapentaplegic; STAT6, signal transducer and activator of transcription 6; TGF‐β, transforming growth factor beta; TGF‐βR, transforming growth factor beta receptor.

Importantly, the strength and COPD‐specificity of the evidence differ between the four myokines. Irisin currently has the most direct evidence linking a myokine to COPD‐related lung pathology, with protective effects demonstrated in cigarette smoke‐induced emphysema and associations between circulating irisin and emphysematous destruction in patients with COPD [121]. MG53 has shown lung‐protective effects across several independent injury models, including experimental emphysema, although direct evidence for pulmonary effects in patients with COPD is lacking [70, 71, 72, 73, 74, 75]. Evidence for apelin is relatively consistent across fibrosis and acute lung injury models but has not been directly established in COPD [59, 60, 61, 62, 63, 64, 65, 66]. The evidence for Metrnl is more recent and similarly remains largely restricted to preclinical models of fibrosis, acute lung injury and allergic airway disease [82, 83, 84, 85, 86]. Thus, although potential biological relevance is supported for all four myokines, direct evidence for a causal role in COPD remains limited.

However, confirmation of such myokine involvement in lung homeostasis would support a concept that skeletal muscle dysfunction, physical inactivity and sarcopenia may aggravate pulmonary disease not only by reducing exercise capacity, but also by disturbing a protective myokine‐mediated axis. More broadly, circulating myokine profiles have been associated with inactivity in patients with stable COPD, supporting a biological relationship between muscle disuse and circulating myokine levels [122]. As skeletal muscle is an important source of myokines such as irisin, apelin, MG53 and Metrnl, the loss of muscle mass characteristic of COPD may reduce the amount of myokine‐producing tissue, thereby limiting the release of these factors into the circulation and their subsequent delivery to the lungs. Direct evidence that circulating levels of the myokines discussed in this review are determined by skeletal muscle mass remains limited and appears to differ between individual myokines. Circulating irisin has been positively associated with muscle mass and strength in some populations, and exercise‐induced changes in irisin have correlated with increases in muscle mass [123, 124]. However, another study found no relationship between circulating irisin and appendicular muscle mass, skeletal muscle mass index or sarcopenia status in older adults [125]. Similarly, circulating apelin was not associated with muscle mass or changes in appendicular lean mass in cross‐sectional and longitudinal cohorts of older adults [126, 127]. Supporting the functional relevance of these associations, circulating irisin is reduced in patients with COPD, including those with COPD‐associated sarcopenia, and has been associated with physical activity and inspiratory muscle strength [128, 129, 130]. Lower circulating irisin is also associated with emphysematous destruction and reduced MG53 levels have been observed in sarcopenic patients with COPD [77, 121, 131]. By contrast, serum Metrnl was positively, although weakly, correlated with appendicular skeletal muscle mass index in older adults [132], while plasma MG53 correlated with skeletal muscle mass index and imaging‐based measures of muscle quantity in patients with COPD [77]. Consequently, reduced myokine availability may impair muscle‐derived support of pulmonary homeostasis and repair, potentially contributing to disease progression. Therefore, myokines may represent a mechanistic bridge between pulmonary rehabilitation, skeletal muscle health and lung preservation in COPD. Interestingly, exercise training has already been shown to increase circulating levels of myokines in COPD, including irisin in a patient cohort and in a murine model of cigarette smoke‐induced COPD [50, 130]. Overall, this evidence suggests that a disturbed myokine pool in COPD may aggravate lung pathology and that exercise interventions might be a suitable strategy to improve lung health in COPD through myokine release by skeletal muscle.

Several important barriers currently limit translation of this concept to clinical practice. Direct validation of these mechanisms in human COPD lung tissue remains limited, and the physiologically relevant circulating concentrations of individual myokines and their ability to reach pulmonary target cells are poorly defined. Specifically, most experimental evidence discussed in this review demonstrates the effects of exogenous myokine administration or experimental overexpression rather than physiological delivery from skeletal muscle to the lung. These studies therefore establish that individual myokines can modulate pulmonary processes, but do not by themselves demonstrate an endocrine skeletal muscle–lung axis. Whether muscle‐derived myokines reach pulmonary target cells at biologically effective concentrations and to what extent endocrine versus exogenously administered myokines contribute to these effects, remains unresolved. Moreover, although the pulmonary effects discussed in this review are predominantly protective, myokines may exert additional effects in extra‐pulmonary tissues that are not covered here. Such effects may be particularly relevant in COPD given the frequent occurrence of systemic comorbidities and should be considered when evaluating strategies aimed at modulating circulating myokine levels. For example, circulating MG53 has been reported to induce systemic insulin resistance, while neutralization of circulating MG53 was protective in diabetic mice; MG53 has also been reported to act as an E3 ubiquitin ligase targeting the insulin receptor and IRS‐1 for degradation, although subsequent studies have questioned a critical role for MG53 in skeletal muscle insulin signaling and glucose homeostasis [69, 133]. In contrast, sustained elevation of circulating MG53 improved tissue regenerative capacity without impairing glucose handling, suggesting that the metabolic consequences of elevated circulating MG53 remain unresolved [134]. Most importantly, no interventional studies have yet demonstrated that selective modulation of an individual myokine improves clinical outcomes in COPD. Establishing physiological exposure ranges, target‐cell engagement and causal effects in human COPD will therefore be essential to determine the clinical relevance of the proposed muscle–lung axis.

## 6 Conclusion

Emerging evidence suggests that skeletal muscle can benefit lung health via the release of myokines with potential implications for chronic respiratory disease. This evidence supports the notion that healthy skeletal muscle and/or exercise may be required to support lung health in COPD, and that reduced muscle mass could contribute to disease progression. In COPD, myokines such as irisin, apelin, MG53 and Metrnl may counteract inflammation, oxidative stress and impaired tissue homeostasis. However, direct evidence for a skeletal muscle‐derived endocrine contribution to lung protection remains limited, and the proposed muscle–lung axis should therefore be considered a plausible but not yet established disease mechanism. Future research should aim to investigate the causal impact of muscle secretome on lung structure and function in (pre)clinical models, identify key mediators and pathways involved in this crosstalk, and explore how exercise‐induced or nutritionally enhanced myokine release could be deployed to restore pulmonary function and integrity.

## Author Contributions

Sven Manse, Ramon C.J. Langen, and Harry R. Gosker conceived the review. Sven Manse performed the literature search and synthesis. Sven Manse drafted the manuscript. Ramon C.J. Langen, Harry R. Gosker, and Irene H. Heijink critically revised the manuscript. Ramon C.J. Langen, Irene H. Heijink, and Harry R. Gosker supervised the work and acquired funding. All authors read and approved of the final manuscript.

## Funding

This work was supported by ZonMw (Netherlands Organisation for Health Research and Development), 09120012010088.

## Conflicts of Interest

The authors declare no conflicts of interest.

## Supporting information


**Supplementary Table S1:** PubMed search strategy used to identify studies investigating pulmonary effects of the selected myokines. All searches were performed in PubMed. Studies primarily focusing on lung cancer were excluded, as malignant lung disease fell outside the scope of this review. No restrictions on publication date were applied. Publications were manually screened for relevance before inclusion in the review.

## Data Availability

Data sharing is not applicable to this article as no datasets were generated or analyzed during the current study.
