# Supplementary material for: Myokines as Support Mechanisms for Lung Maintenance and Repair: Implications for Chronic Obstructive Pulmonary Disease
Source: FASEB J. 2026 Nov 14;40(22):e72476. doi: 10.1096/fj.202601931RRRR (PMC13768830; doi:10.1096/fj.202601931RRRR)
Supplement: Supplementary file 1 [file FSB2-40-e72476-s001.docx]

| **Myokine** | **PubMed search string** | **Search date** |
| --- | --- | --- |
| **Irisin** | ((Irisin[Title]) OR (FNDC5[Title])) AND ((lung*[Title/Abstract]) OR (pulmo*[Title/Abstract]) OR (airway*[Title/Abstract]) OR (respiratory[Title/Abstract]) OR (alveo*[Title/Abstract]) OR (bronch*[Title/Abstract])) | 01 March 2026 |
| **Apelin** | (Apelin[Title]) AND ((lung*[Title/Abstract]) OR (pulmo*[Title/Abstract]) OR (airway*[Title/Abstract]) OR (respiratory[Title/Abstract]) OR (alveo*[Title/Abstract]) OR (bronch*[Title/Abstract])) | 01 March 2026 |
| **MG53** | ((MG53[Title]) OR (Mitsugumin 53[Title]) OR (Mitsugumin-53[Title]) OR (TRIM72[Title])) AND ((lung*[Title/Abstract]) OR (pulmo*[Title/Abstract]) OR (airway*[Title/Abstract]) OR (respiratory[Title/Abstract]) OR (alveo*[Title/Abstract]) OR (bronch*[Title/Abstract])) | 01 March 2026 |
| **Metrnl** | ((Metrnl[Title]) OR (Meteorin-like[Title]) OR (Meteorin-beta[Title]) OR (cometin[Title]) OR (subfatin[Title]) OR (IL-41[Title])) AND ((lung*[Title/Abstract]) OR (pulmo*[Title/Abstract]) OR (airway*[Title/Abstract]) OR (respiratory[Title/Abstract]) OR (alveo*[Title/Abstract]) OR (bronch*[Title/Abstract])) | 01 March 2026 |

**Supplementary Table S1. PubMed search strategy used to identify studies investigating pulmonary effects of the selected myokines.** All searches were performed in PubMed. Studies primarily focusing on lung cancer were excluded, as malignant lung disease fell outside the scope of this review. No restrictions on publication date were applied. Publications were manually screened for relevance before inclusion in the review.
